# Inductive Coupling of Bipolar Signals with a Conjugate Coil Pair for an Analog Passive ECG Sensor Using a PPy-Coated pvCNT Dry Electrodes

**DOI:** 10.3390/s23115283

**Published:** 2023-06-02

**Authors:** Mohammad Abu-Saude, Bashir I. Morshed

**Affiliations:** 1 Department of Electrical and Computer Engineering, University of Memphis, Memphis, TN 38152, USA; abusaude@gmail.com; 2 Department of Computer Science, Texas Tech University, Lubbock, TX 79409, USA

**Keywords:** body-worn sensor, carbon nanotube, dry ECG electrode, ECG signal capture, passive sensor, wireless sensor, WRAP sensor

## Abstract

The wireless capture of analog differential signals from fully passive (battery-less) sensors is technically challenging but it can allow for the seamless capture of differential biosignals such as an electrocardiogram (ECG). This paper presents a novel design for the wireless capture of analog differential signals using a novel conjugate coil pair for a wireless resistive analog passive (WRAP) ECG sensor. Furthermore, we integrate this sensor with a new type of dry electrode, namely conductive polymer polypyrrole (PPy)-coated patterned vertical carbon nanotube (pvCNT) electrodes. The proposed circuit uses dual-gate depletion-mode MOSFETs to convert the differential biopotential signals to correlated drain-source resistance changes and the conjugate coil wirelessly transmits the differences of the two input signals. The circuit rejects (17.24 dB) common mode signals and passing only differential signals. We have integrated this novel design with our previously reported PPy-coated pvCNT dry ECG electrodes, fabricated on a stainless steel substrate with a diameter of 10 mm, which provided a zero-power (battery-less) ECG capture system for long duration monitoring. The scanner transmits an RF carrier signal at 8.37 MHz. The proposed ECG WRAP sensor uses only two complementary biopotential amplifier circuits, each of which has a single-depletion MOSFET. The amplitude-modulated RF signal is envelope-detected, filtered, amplified, and transmitted to a computer for signal processing. ECG signals are collected using this WRAP sensor and compared with a commercial counterpart. Due to the battery-less nature of the ECG WRAP sensor, it has the potential to be a body-worn electronic circuit patch with dry pvCNT electrodes that stably operate for a long period of time.

## 1. Introduction

Biopotential signals such as the electrocardiogram (ECG/EKG), electroencephalogram (EEG), electromyogram (EMG), and galvanic skin response (GSR) require impedimetric interfacing with skin. Theses neurophysiological signals play vital roles in disease diagnosing, patient monitoring, and non-clinical applications [1]. Traditional data collection requires wired sensors which limits comfort and ease of usability. The demand for using alternatives for continuous and user-friendly monitoring has increased, especially in situations in which physical wire connections are unsuitable [2]. Furthermore, the conventional gel/wet electrodes only operate well on a short-term basis due to evaporation when exposed to the environment and the subsequent reduction in conductivity [3].

Dry electrodes are a promising alternative to traditional wet electrodes as they are easy to use and operate for long durations without degradation of impedance. We have reported a novel dry interfacing electrode using patterned vertically aligned CNTs (pvCNT) coated with a conductive polymer (PPy), which is mechanically stable and additionally has very low and stable impedance [4]. MWCNTs are used due to their electrical conductivity [5]. Moreover, pvCNTs provide a means for achieving good contact over rough and hairy skin surfaces.

Traditional biopotential measurement requires complex electronics, a power source, and wires which increase the cost and size of the device, as well as reducing its usability. This can be a challenge in situations where wired connections are impractical such as in body-worn or implantable applications [2]. Passive wireless sensing is an alternative method to measuring bioelectric signals by which the device is highly simplified, battery-less (zero-power), and no physical connection is required. Therefore, continuous patient monitoring is more practical and can be used in body-worn sensors to collect physiological signals unobtrusively [6,7,8]. In 1967, the first passive wireless sensor was developed for measuring intraocular pressure by implanting a pair of spiral coils inside the eye [9]. Furthermore, a similar type of resonator was used to measure intracardiac blood pressure [10,11] using a capacitive pressure sensor. ECG signals were measured using a wireless radio system based on variable capacitive diodes (varactors) as the frequency shifting component in the resonance circuit [12], but the system requires a battery. Similarly, a varactor has been used as a capacitive component with a passive LC resonator for measuring ECG, whereby biopotential electrodes were connected over the varactor ends [13]. The change in the varactor capacitance induced by the biopotential signal can be measured over an inductive link as a modified reflected impedance. A geophone-based sensing system for extracting electrocardiogram (ECG) signals was described in [14], using heart beat vibration through a bed mattress. Two ECG electrodes have also been tested with minimal hardware complexity [15]. However, physical contact is required for measurements.

We have previously developed wireless resistive analog passive (WRAP) sensors [16,17] that can transmit analog signals without a battery (fully passive) and do not contain any digital chips. This provides a novel ability to collect body signals that requires bodily contact with electrodes (such as ECG/EKG). Our proposed system consists of a battery-less electronic patch wireless sensor (WRAP sensor with a secondary coil) and a wearable wireless data collection device (scanner with a primary coil) utilizing the inductive loading principle. The novel WRAP sensor is based on an LC resonance that is inductively coupled between two printed spiral coils (PSCs). Various physiological signals have been captured such as respiration rate, heart rate, and core body temperature [16,17,18,19,20].

However, for ECG signal measurement, the differential inputs of two ECG electrodes are required. The differential amplifier rejects the common mode signal and amplifies the differences between the two signals. In this work, we have designed a novel WRAP sensor for measuring differential biopotential signals using a conjugate spiral coil pair: a primary coil (i.e., the scanner responsible for transmitting the RF signal and performing the readout) and a secondary coil (i.e., the sensor causing the modulation). The primary and secondary coil designs have been studied and optimized by our research group previously [18]. Two depletion-mode N-channel dual-gate MOSFETs are used to connect the differential inputs that change the loading of the two secondary coils according to the ECG signals. As a result, only the differential biopotential signals are transmitted over the conductive link by modulating the carrier signal while the common signals of both sensor coils are canceled out. The system is integrated with dry pvCNT electrodes that are attached to the body. In this study, we used a wire to attach the sensor to the electrodes for experimental purposes; however, the sensor could be used as a standalone or implanted sensor to measure any differential biopotential signals. The key contributions of this study are the following:

- The feasibility of using a two-part system in which the part that attaches to the body does not require any battery (zero-power) while the other part wirelessly powers and probes the body-worn part to collect ECG data;
- Integration of the ECG electrode (zero-power) with pvCNT dry electrode technology so that data can be collected over a very long time without the need to replace the electrodes.

## 2. Hardware Description

### 2.1. PvCNT Electrode

**Figure 1:**
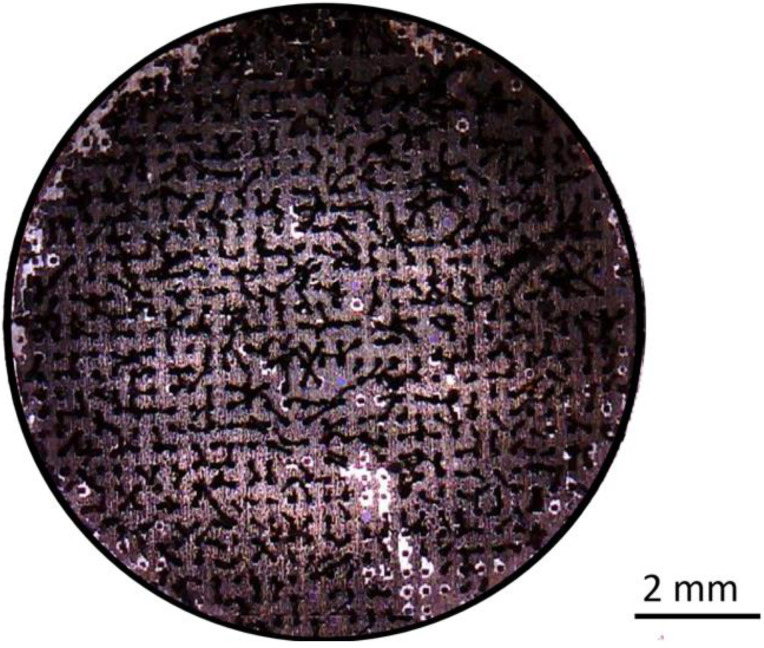
An optical image of a PPy-coated pvCNT electrode.

The pvCNT electrodes were developed with MWCNTs grown in a patterned vertically aligned shape on a stainless steel substrate (ϕ = 10 mm, 2 mm thick), forming a bristle-like structure [21,22]. The pillars were grown on 100 µm squared bases with heights between 1 and 1.5 mm and a distance between each pillar of 200 µm in an array formation. This structure provides the capability of measuring signals through hairy and rough areas of the skin. The pvCNTs were fabricated using a chemical vapor deposition (CVD) system. Figure 1 shows an optical image of a polypyrrole-coated pvCNT electrode.

### 2.2. Planar Wireless Coil Design

An essential parameter of a measurement system design is the quality and the strength of the link (i.e., the inductive coupling) between the primary and secondary coils. The dimensions of the coil antennas depend on the operating frequency. In this work, we used 8.27 MHz; thus, the size of the antennas would be suitable for body-worn sensors. The planar design was chosen such that the printed circuit boards (PCBs) would be easy to construct, have a small size, and provide accurate results. Thus, the inductive coils are designed as PSC antennas on custom-designed PCBs.

**Figure 2:**
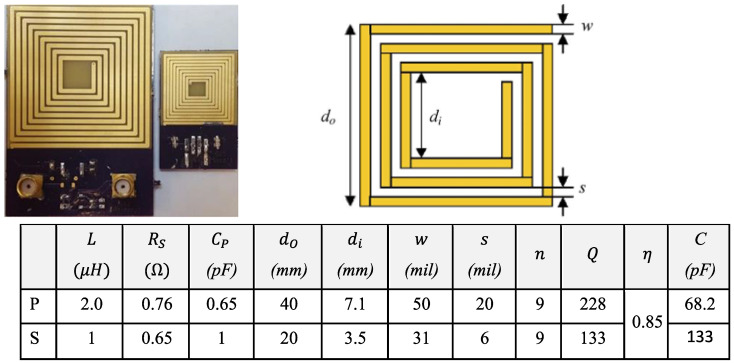
The selected coil design and its specifications (table adapted from [18]).

One of the advantages of using rectangular coil cross-section results from the skin effect occurring in conductors is that the AC current is better with a higher surface area at a high frequency [23]. The coupling coefficient between the spiral antennas would be optimized if both were placed and aligned coaxially. However, PSC antennas can be more tolerant to misalignment [24]. The coupling coefficient k is a convenient way to specify the coupling efficiency between two inductors and is defined by the following:

(1) $$k=\frac{M}{\sqrt{L_{1}L_{2}}}$$

where *M* is the mutual inductance, and *L_1_* and *L_2_* are the self-inductances of the two coils. When mutual inductance increases, the coupling coefficient also increases. The mutual inductance becomes greater as the diameter of the coils increases. The effect of this phenomenon was reported by our research group elsewhere [18]. The relationship between the size of the coils and the distance between those coils is presented in (2) [25]:

(2) $$d=\frac{1}{2}\sqrt{d_{p}-d_{s}}$$

where *d_p_* and *d_s_* are the diameters of the primary and secondary coils, respectively, and d represents the coaxial distance. Optimal coupling can be achieved by making the diameter of the primary coil greater than the diameter of the sensor coil. The inductive link coupling coefficient is dependent on distance and alignment, such as lateral shift or the angular orientation of the secondary coil with respect to the primary, which was explored in our past study [26]. Our research group conducted an intensive study on an iterative method to find the optimal coil design which will therefore be left undiscussed in this context. Figure 2 shows the selected design and the list of parameters for optimal efficiency as reported earlier [18]. The table is adapted from [18] and includes the parameters of the primary (P) and the secondary (S) coils, where L, R_S_, and C_P_ are the equivalent components for each coil, n is the number of turns, Q is the quality factor, C is the matching capacitor, and η is the efficiency.

Cadence Allegro (Cadence Design Systems Inc., San Jose, CA, USA) was used to design the printed circuit boards (PCBs). The outer diameters of the primary and the secondary coils are 40 and 20 mm, respectively.

### 2.3. Measurement System

**Figure 3:**
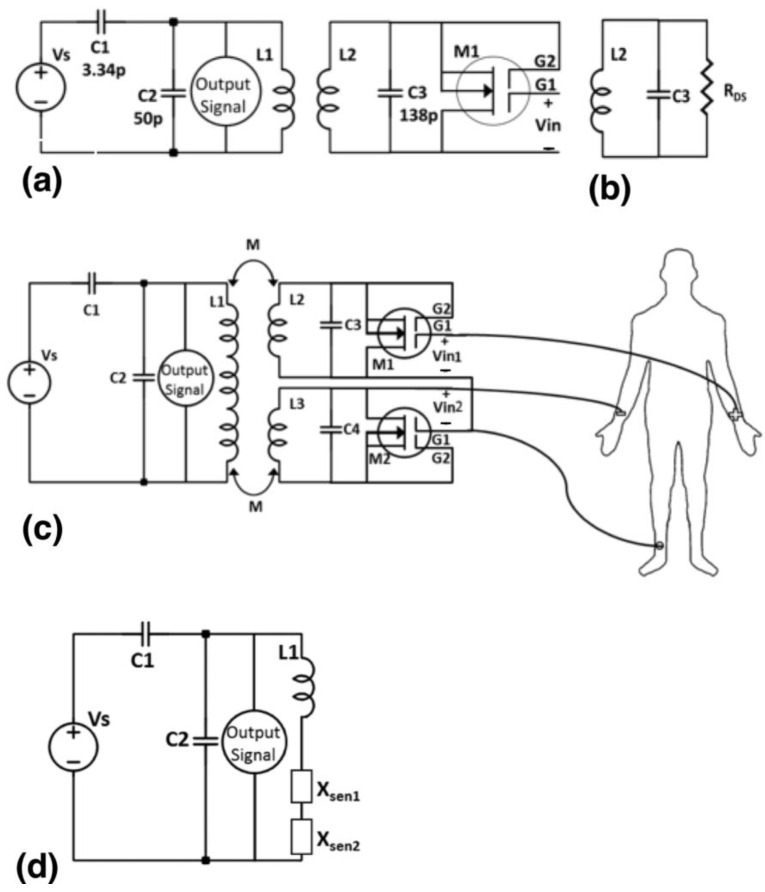
(**a**) Schematic of the primary and secondary coils for biopotential sensing with a BF908R depletion-mode N-channel dual-gate MOSFET. (**b**) The equivalent secondary circuit depicting the MOSFET drain-source resistance as R_DS_. (**c**) Two WRAP sensors are used for the sensing of the differential input voltages to access ECG signals from a human body. (**d**) The sensor impedances are represented by equivalent impedances, X_sen1_ and X_sen2_.

A schematic diagram of a simplified scanner circuit and a WRAP sensor for biopotential signal access is presented in Figure 3a. The equivalent circuit for the WRAP sensor is a parallel combination of RLC as shown in Figure 3b. The ECG WRAP sensor proposed here consists of three PSC coils, where two identical conjugate coils (*L_2_* and *L_3_*) are used on the sensor side and one primary coil (*L_1_*) is used on the scanner side as depicted in Figure 3c. The primary coil is coupled to the two sensor coils through an inductive link. The overall circuit can be reduced to a single equivalent circuit diagram with reflected impedances X_sen1_ and X_sen2_ of the sensors, as shown in Figure 3d.

We have previously reported biopotential capture with single-gate enhancement and depletion-mode MOSFETs [17,20]. However, single-gate MOSFET amplifications were insufficient in collecting ECG signals with inductive coupling. After intensive research to resolve this technical challenge, a dual-gate MOSFET with the Figure 3a configuration was found to achieve a sufficiently high and stable gain. Here, we report ECG signals captured using N-channel dual-gate MOSFETs (depletion-mode), which were selected due to their high forward transfer admittance for small input voltages (Vin) at gate 1. These MOSFETs have a thin layer of N-type silicon placed below the gate, which makes it normally ON. Furthermore, the input resistance of the MOSFET transistor is very high; hence, the depletion-mode MOSFET is very well suited for its use as a voltage-controlled resistance for biopotential measurements. The input voltage can be correlated to the resistive variation in drain-source resistance of the MOSFET (R_DS_). Two SMA connectors on the scanner board provided the carrier input, an RF signal of 8.37 MHz, and access to the coil L1 voltage, which carried the modulated signal. The capacitors C1 and C2 were used to match the 50 Ω antenna and adjust the resonance frequency with L1, respectively. The sensor boards are identical and they have matching circuit component values (C3 and C4) which are used to tune the antennas to the same resonance frequency. Gate 2 of the MOSFETs M1 and M2 is connected to the coupled signal (at the source). Three electrodes are connected to the human body (e.g., to capture an ECG signal) as shown in Figure 3c.

**Figure 4:**
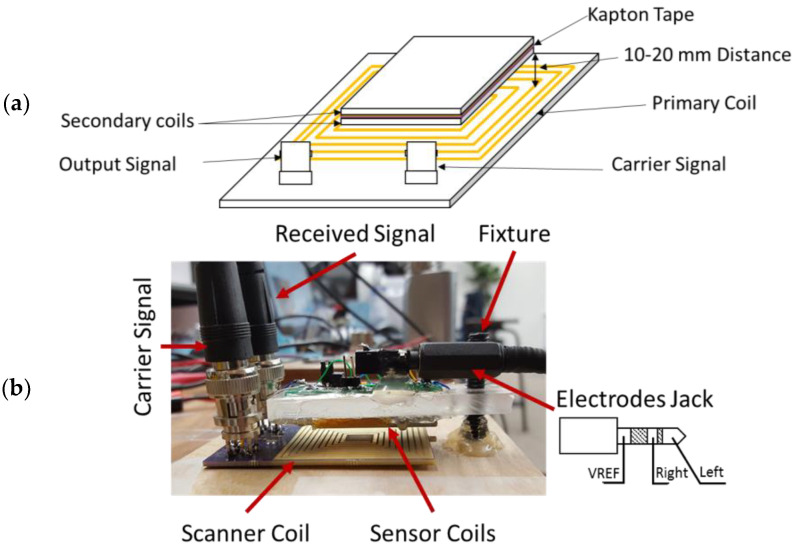
(**a**) Diagram depicting the conjugate coil (separated by Kapton tape) and the scanner coil positioned in a co-axial position. (**b**) A photograph of the setup used for the scanner and the sensors to capture signals in the differential mode.

The planar coils of the sensor boards are placed to face each other and Kapton tape is used as an insulation material as shown in Figure 4a. A fixture is used to keep the boards in a parallel co-axial position as seen in Figure 4b. The distance between the primary and the secondary coils is 10–20 mm.

## 3. Theory of Operation

The wireless WRAP sensors (with secondary coils) inductively coupled to the scanner (primary) coil have impedances, *Z_2_* and *Z_3_*, as given below [27]:

(3) $$Z_{2}=\left(j\omega L_{2}+\left(\frac{1}{j\omega C_{3}}\right)||\;R_{DS1}\right)$$

(4) $$Z_{3}=\left(j\omega L_{3}+\left(\frac{1}{j\omega C_{4}}\right)||\;R_{DS2}\right)$$

where *R_DS_*_1_ and *R_DS_*_2_ are the corresponding drain-source resistances of MOSFET 1 (*M1*) and MOSFET 2 (*M*2), respectively, of the two conjugate sensors. As shown in Figure 3d, the impedance of each sensor can be reduced to a single impedance component on the primary side as given in Equation (5) by applying Kirchhoff’s theorem separately to the primary and secondary circuits:

(5) $$X_{sen}=\left(\;\frac{{\left(\omega M\right)}^{2}}{Zsen\left(\omega \right)}\right)$$

where *ω* is the angular frequency of the induced magnetic field and *M* represents the mutual inductance between the primary and secondary coil pair. Finally, assuming that the mutual inductances between the primary and two secondary coils are equivalent yields the following: *X_sen_*_1_ = *X_sen_*_2_ = *X_sen_*.

The currents through these sensors, *I_s_*_1_ and *I_s_*_2_, are dependent on the gate-source voltages of these two MOSFETs. The input voltages are connected between the gate and the drain of each MOSFET, therefore:

(6) $$V_{in1}=+\left(V_{GS1}-V_{DS1}\right)$$

(7) $$V_{in2}=-(V_{GS2}-V_{DS2})$$

These MOSFETs are normally ON and there is an initial current in each sensor’s circuit and any change in input voltages will change *I_s_*_1_ and *I_s_*_2_ in the opposite way. These sensor currents, in addition to the primary current (*I_p_*), dictate the voltage across the primary coil (*V_p_*) as given in the following:

(8) $$V_{p}=j\omega L_{1}I_{p}-j\omega M(I_{s1}+I_{s2})$$

Here, *M* is the mutual inductance as given in (1). Equation (8) represents an expression where the primary voltage is dependent on the currents of the sensors. If *V_in1_* and *V_in2_* are connected together (common-mode signal), the sum of the current will remain constant and there will be no change in *V_p_.* So, the main voltage *V_p_* will be modulated and changed only when *V_in_*_1_ and *V_in_*_2_ are different. Thus, the proposed coil sensor only collects differential mode signals and suppresses common mode signals.

## 4. Data Collection

**Figure 5:**
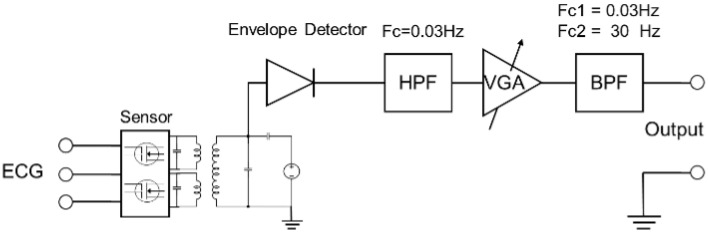
Block diagram of the ECG signal capture process and analysis.

The biosignal modulates the impedance of the resonance circuit, which in turn modulates the carrier signal. By demodulating this modulated signal with an envelope detector, the ECG signal in the sensing unit can be detected. The received signal was extracted using an envelope detector followed by a unity gain voltage follower with a high pass filter, a variable gain amplifier, and a bandpass filter with *f_c_*_1_ = 0.03 Hz and *f_c_*_2_ = 30 Hz, as shown in Figure 5. The output is connected to an oscilloscope (Model: MDO3024; Tektronix Inc., Santa Clara, CA, USA) and the data from the oscilloscope were saved in .csv format. A signal generator (Model: DG4162; Rigol Technologies Inc., Beijing, China) was used to generate the RF signal to probe the passive sensors. This data was later compiled, analyzed, and plotted in MATLAB (MathWorks Inc., Natick, MA, USA).

### 4.1. Bench Test Experiment Setup

Two test bench experiments were conducted to test the sensitivity of the depletion-mode MOSFET and the functionality (i.e., differential mode) of the sensor. In the first experiment, only one MOSFET was used as shown in Figure 3a. The voltage was applied through a BNC attenuator to provide microvolt range inputs. In the other experiment, the complete ECG WRAP sensor was set up in conjugate coil pair formation with two applied input voltages *V_in_*_1_ and *V_in_*_2_. Three function generators (Model: DG4162; Rigol Technologies Inc., Beijing, China) were used in this setup, one for the carrier frequency and the other two for each of the input voltages. All the ground terminals of the secondary circuit were different than the ground of the primary circuit.

### 4.2. ECG Measurement Setup

**Figure 6:**
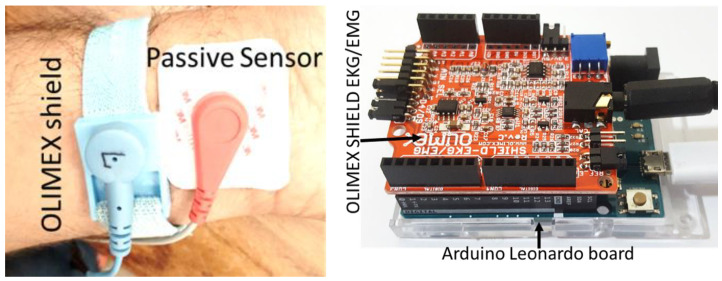
ECG electrode setup for signal capture (**left**) and EKG/EMG shield from OLIMEX connected on an Arduino Uno board (**right**).

In the first ECG measurement, the performance of the passive sensor was further investigated and compared to the quality of the recorded ECG signal with the open-source commercial hardware OLIMEX EKG/EMG shield (OLIMEX Ltd., Plovdiv, Bulgaria) as shown in Figure 6 (right). In vivo ECG data were captured using commercial gel-type ECG electrodes Ag/AgCl (Model: GS-26; Bio-Medical Instruments, Clinton Twp, MI, USA) for the passive sensor and wrist electrodes for the EKG/EMG shield as shown in Figure 6 (left). The left arm was connected to *V_in_*_1_, the right arm, to *V_in_*_2_, and the right leg, to the reference point as shown in Figure 3c. The shield EKG/EMG converted the analog differential signal attached to CH1_IN+/CH1_IN– inputs into a single stream of data as the output. The output signal was connected directly to the oscilloscope. All the ECG measurements were taken at the same time by both sensors.

### 4.3. ECG with pvCNT Measurement Setup

**Figure 7:**
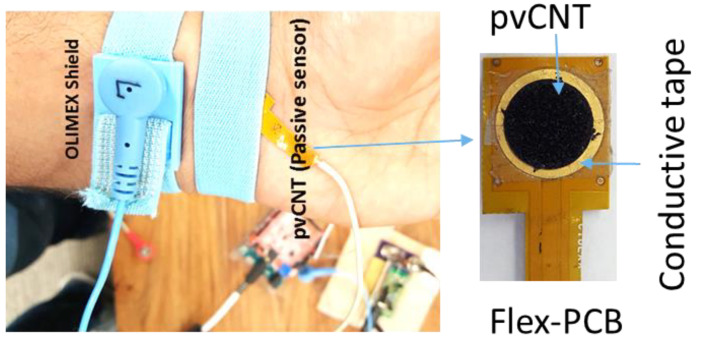
The pvCNT setup and the OLIMEX dry electrode attached to the left arm.

Another set of ECG measurements was performed using a pvCNT electrode. The pvCNT electrode was attached to a flexible PCB (flex-PCB) with double-sided z-axis conductive tape (Electrically Conductive Adhesive Transfer Tape 9703; 3M Company, St. Paul, MN, USA), as shown in Figure 7 (right), on an exposed Cu pad with a diameter of 10 mm in order to provide a low impedance. The flex-PCB was custom-designed and prototyped via Cirexx Intl., Santa Clara, CA, USA. Three electrodes were placed on the human body at the following locations: left forearm, right forearm, and right leg. All of the measurements of the ECG signals were conducted at room temperature with no skin preparation. The pvCNT was attached to the hand using a rubber band as shown in Figure 7 (left).

## 5. Experimental Results

### 5.1. Bench Test Results

**Figure 8:**
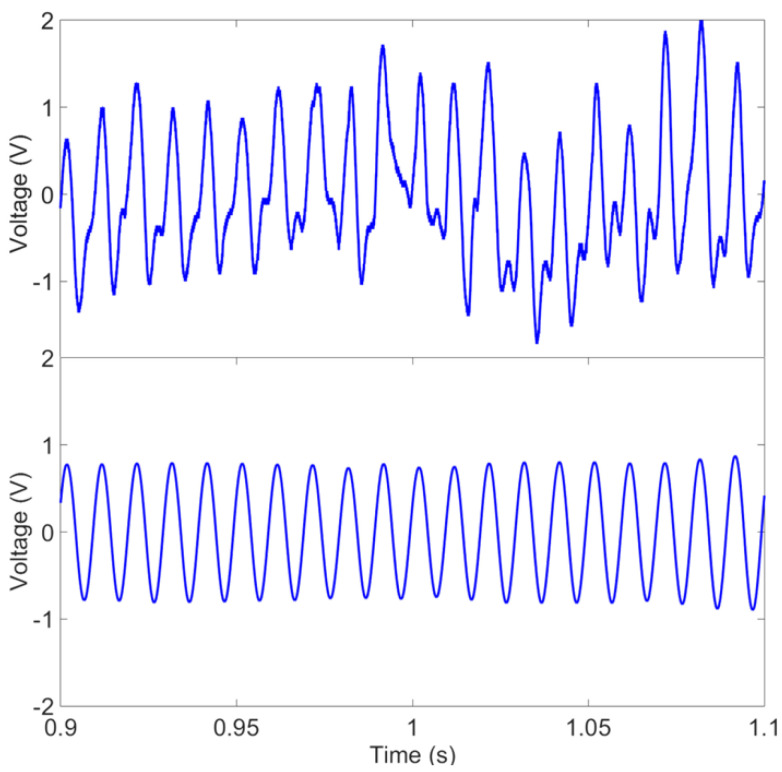
The wirelessly captured signal with a 100 µV input voltage. The raw (**top**) and filtered data (**bottom**) are plotted.

In the first set of experiments, a microvoltage using a BNC attenuator of 40 dB was applied at low frequencies to meet the requirement of measuring biopotential signals. The setup is able to apply signals as low as 40 µV. Figure 8 shows the received signal of 100 µV at a frequency of 100 Hz before and after filtering the collected signal. The signal was captured and processed using MATLAB.

**Figure 9:**
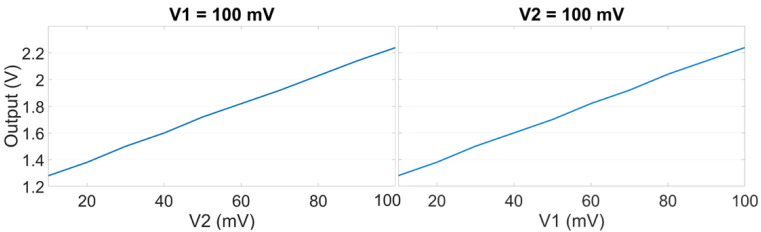
The response of the differential sensor when one input was fixed and the other was changed.

The experiments using the differential detection mode setup were conducted to measure the CMRR for the differential sensor. For this experiment, the received signal was connected directly to a unity gain low pass filter to remove the carrier signal. The average common (A_CM_) and differential (A_DM_) mode gains were 0.1703 and 1.24, respectively; therefore, the calculated CMRR was 17.24 dB. Figure 9 shows a linear response of the sensor when one input was fixed to a voltage and the other input voltage was linearly changed.

**Figure 10:**
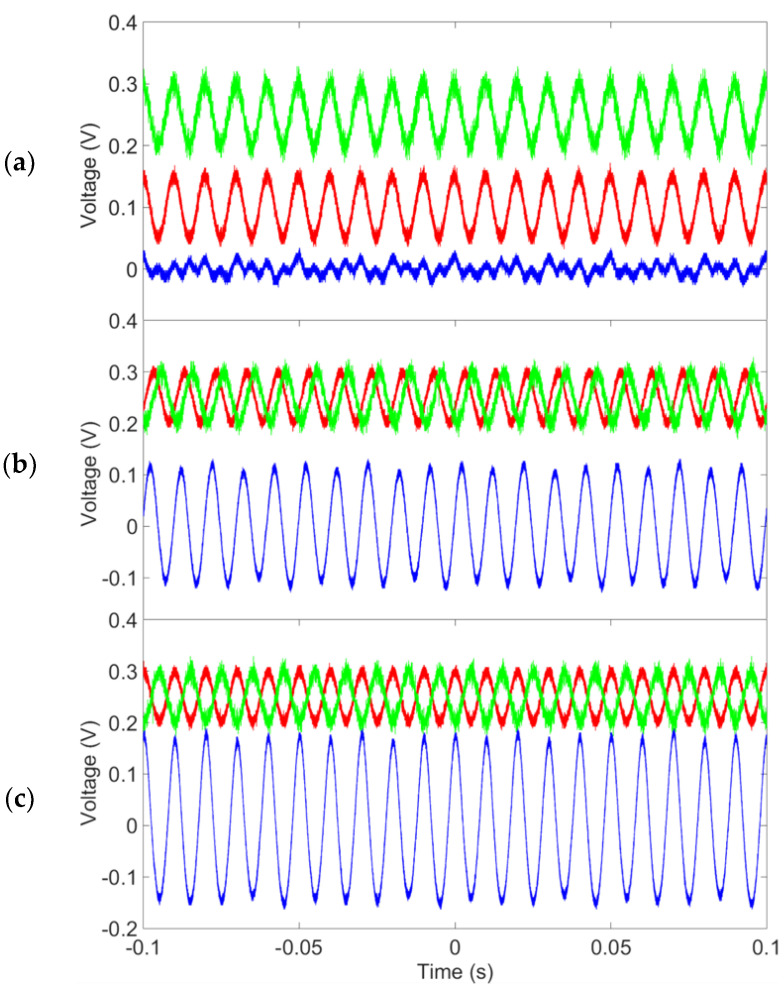
The output of the differential and common mode setup. The phase differences between V_in1_ and V_in2_ were the following: (**a**) 0°, (**b**) 90°, and (**c**) 180°.

Figure 10 shows the received signal (blue) as the phase between the *V_in_*_1_ (green) and *V_in_*_2_ (red) as equal to 0°, 90°, and 180°, respectively. Both *V_in_*_1_ and *V_in_*_2_ are sinusoidal signals with peak-to-peak voltages of 100 mV and frequencies of 100 Hz. The results show that the output signal was significantly attenuated when both input signals were in-phase (common mode), while the output signal was amplified when they were out-of-phase (differential mode).

### 5.2. ECG Measurement Results

**Figure 11:**
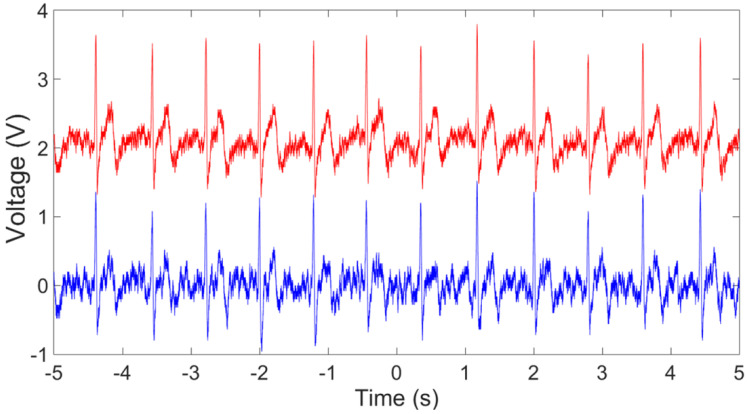
The raw data of the recorded ECG signals using an EKG/EMG shield (red) and the passive ECG WRAP sensor (blue) for 10 s.

**Figure 12:**
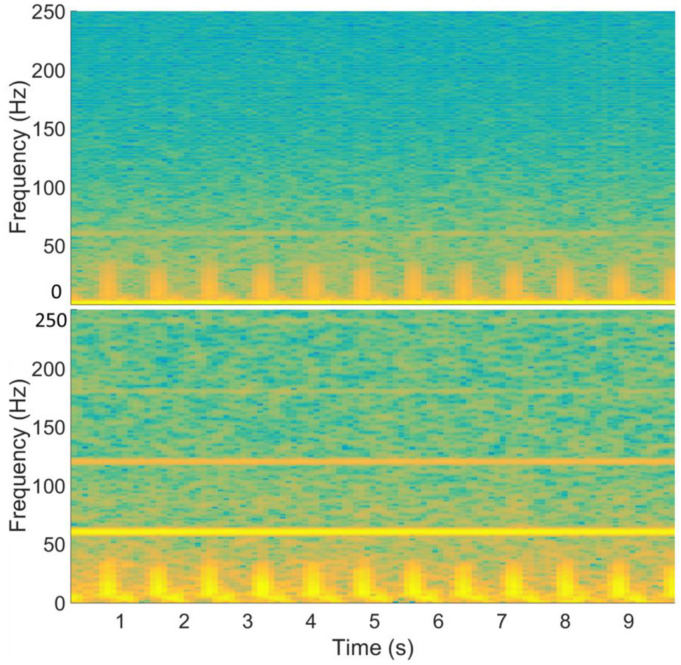
The time–frequency analysis of ECG signals using an EKG/EMG shield (**top**) and the passive ECG WRAP sensor (**bottom**).

**Figure 13:**
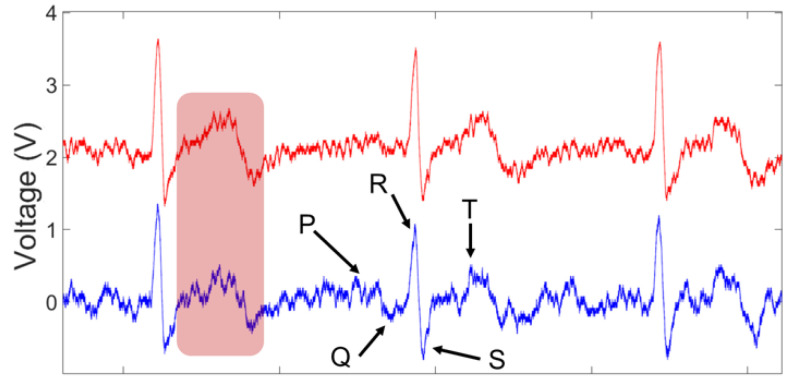

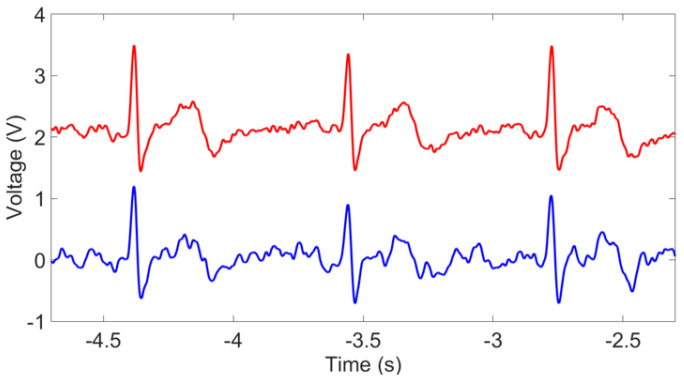
The ECG components (R peaks, QRS complexes, T waves, and P waves) (**top**) and the filtered ECG signal (**bottom**). ECG signals were obtained using an EKG/EMG shield (red) and the passive sensor (blue).

The setup in Figure 3c was used for ECG signal measurement, where the reference of input voltages was connected together and then connected to the right leg. Figure 11 shows the raw data of the ECG signal recorded using the EKG/EMG shield (red) and the passive sensor (blue) for 10 s (100 k points at a sampling frequency of 10 kSPS). Higher noise is observed in the unfiltered signal in the time–frequency analysis plot (Figure 12) for the ECG WRAP data and EKG/EMG shield data (top) and the passive ECG WRAP sensor (bottom). The ECG signals recorded using the WRAP sensor have more utility line noise (60 Hz) and harmonics. A limitation of the ECG WRAP sensor circuit is that there is no filter on the sensor as it is battery-less. However, critical ECG components (R peaks, QRS complexes, T waves, and P waves) are easily recognizable from the raw data without any downstream processing as shown in Figure 13 (top). Further processing with a low pass filter to the signal using MATLAB for both signals produced low noise results, as shown in Figure 13 (bottom).

### 5.3. ECG with pvCNT Measurement Results

**Figure 14:**
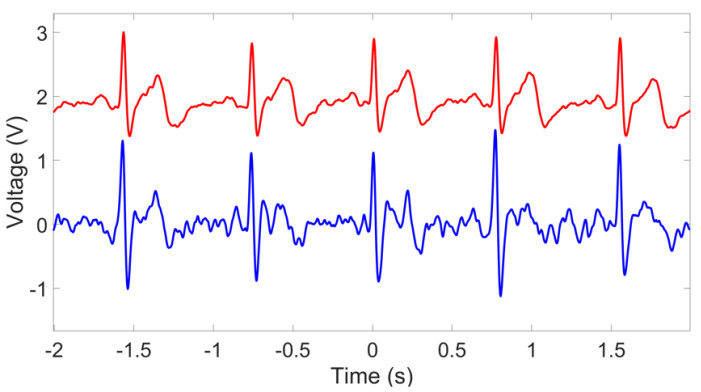
The filtered ECG signal using an EKG/EMG shield (red) and the passive sensor (blue).

Figure 14 shows the ECG signals acquired using the PPy-coated pvCNT dry electrodes. Although the collected ECG signals using pvCNT have higher noise, the typical ECG signal characteristic components, including the P wave, T wave, and QRS complex are still identifiable and observed as the P wave comes before the QRS complex and the T wave comes after. The heart rate, in this case, was 77.8 beats per minute.

**Figure 15:**
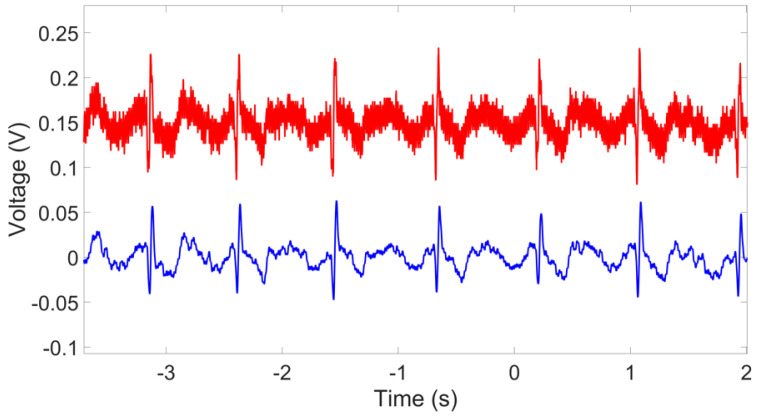
The red line represents the raw data of the recorded ECG signals using pvCNT with the passive sensor and the blue line is the filtered signal.

Figure 15 shows the raw and filtered ECG signal acquired using the pvCNT PPy-coated electrode and bypassing the envelope detector that improves the frequency response, but leading to noisier signals. However, the filtered signal shows that the ECG signal can be identified from the collected signals with this approach as well.

## 6. Conclusions

A novel, wireless, fully passive (battery-less) ECG WRAP sensor and scanner device to measure biopotentials is presented and shown to be able to measure ECG signals from the human body. The novel conjugate coil pair configuration of WRAP sensors uses two WRAP sensors, each utilizing a dual-gate depletion-mode MOSFET for measuring ECG signals from limb lead configuration. The system is successfully able to capture potentials as low as 40 µV, in addition to the ECG signals in vivo. Moreover, in previous works, we have shown the feasibility of using a novel PPy-coated dry electrode (pvCNT) for physiological and neurological bioelectric or impedimetric signal capture [4,22]. In this work, we integrated both the passive wireless device with PPy-coated pvCNTs and demonstrated its ability to measure ECG signals. The results show the promise of developing a battery-less ECG WRAP sensor integrated with a dry CNT-based electrode (pvCNT) that can be worn, or even possibly implanted, and would be suitable for continuous ECG recording over a long duration of time. The scanner, embedded in a vest or other garment, can collect data from the fully passive sensor and transmit it to an edge-computing device (such as smartphones) via Bluetooth for monitoring and analysis. In future work, the data could be wirelessly transmitted to a nearby smart device or a receiver using WiFi or Bluetooth, for example, using an Arduino IoT or Arduino Nano IoT device. Additionally, we will include an edge computing artificial intelligence (AI) algorithm for real-time data processing and classification.

## Data Availability

Data sharing not applicable.
